# Diagnosis and treatment of hepatocellular carcinoma with pelvic metastasis expressing AFP: a case report

**DOI:** 10.3389/fonc.2024.1489725

**Published:** 2025-01-07

**Authors:** Legang Huang, Zijie Wang, Jiankuan Lu

**Affiliations:** Department of Vascular Intervention, Shengli Oilfield Central Hospital, Dongying, Shandong, China

**Keywords:** hepatocellular carcinoma (HCC), alpha-fetoprotein (AFP), HCC metastasis, radioactive seed implantation, PET-CT

## Abstract

This report presents the case of a 68-year-old female patient with hepatocellular carcinoma (HCC) who experienced persistently elevated alpha-fetoprotein (AFP) levels following resection of the primary liver tumor. The patient had previously undergone transcatheter arterial chemoembolization (TACE) and liver tumor resection, but postoperative AFP levels continued to rise, suggesting the possibility of extrahepatic metastasis. PET-CT scans revealed an irregular soft tissue mass in the recto-uterine pouch, which was later confirmed as a HCC metastasis through needle biopsy. The patient subsequently received radioactive seed implantation therapy, leading to a significant decrease in AFP levels. This case highlights the rarity of isolated pelvic metastasis in HCC patients and underscores the importance of AFP in postoperative monitoring. The combination of PET-CT imaging and pathological biopsy is instrumental in improving the detection rate of HCC metastases, enabling more accurate treatment planning for patients.

## Introduction

**Figure 1:**
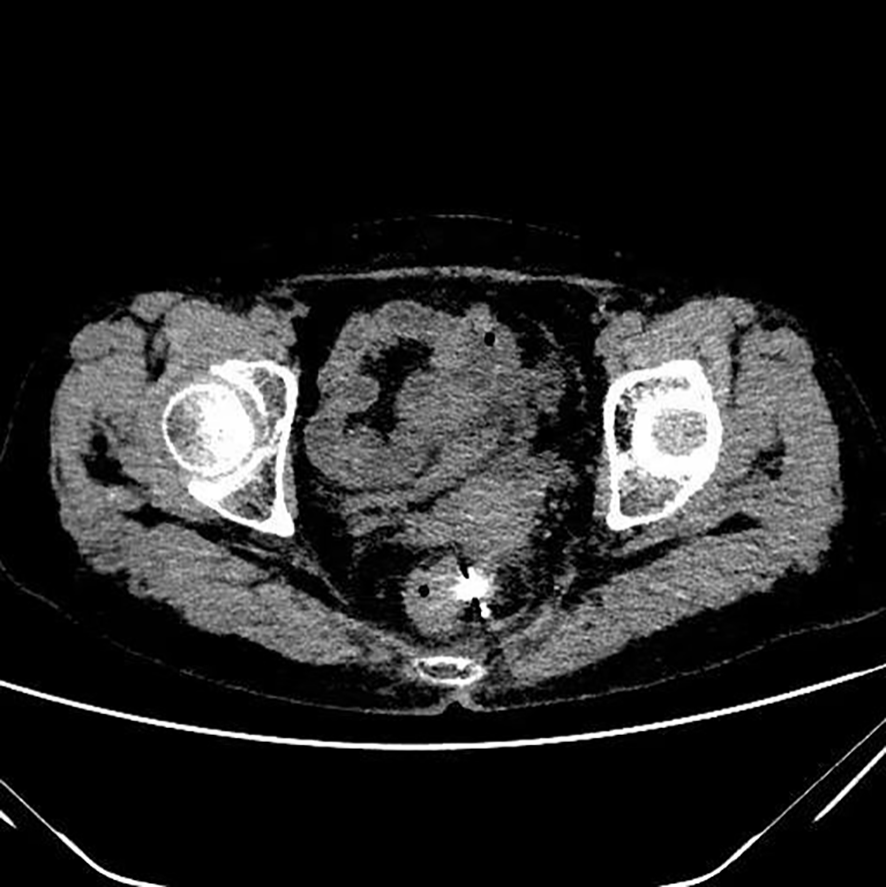
Postoperative pelvic CT image of the patient showing a solid mass in the recto-uterine pouch, with hyperdense radioactive seed implants visible within the lesion.

Hepatocellular carcinoma (HCC) is a malignant tumor with high incidence and mortality rates, making early screening and diagnosis crucial for improving patient prognosis (1). Over the years, the serum biomarker alpha-fetoprotein (AFP) has been widely used in the screening and early warning of HCC due to its advantages of convenient sample collection, minimal invasiveness, and high reproducibility (2). AFP is not only a commonly used marker for diagnosing HCC but also an important tool for monitoring therapeutic efficacy. In clinical practice, AFP has been extensively employed for HCC screening, early diagnosis, postoperative efficacy evaluation, and long-term follow-up monitoring (3, 4). Particularly, when serum AFP levels reach or exceed 400 µg/L, after excluding factors unrelated to HCC such as pregnancy, chronic or active liver disease, gonadal embryonic tumors, and other gastrointestinal tumors, there is a strong indication of the possibility of HCC. For patients with mildly elevated AFP levels, combining imaging examinations with dynamic observations and comparing these with changes in liver function can effectively improve diagnostic accuracy (5, 6). However, literature reports indicate that metastasis is one of the main biological characteristics of HCC and is a leading cause of poor prognosis in patients (7, 8). Distant metastasis in HCC typically indicates advanced disease, significantly increasing the difficulty and complexity of treatment. This report presents a case of a patient with recurrent elevation of AFP levels following resection of the primary HCC lesion, who was ultimately diagnosed with pelvic metastasis through imaging and pathological examinations. After a comprehensive evaluation, the patient received radioactive seed implantation therapy, resulting in a remarkable therapeutic effect. (**Figure 1**) Radioactive seed implantation was chosen for this case due to its high precision in delivering sustained radiation directly to the tumor tissue while sparing surrounding healthy structures. This minimally invasive approach is particularly suitable for patients with anatomically complex lesions, such as pelvic metastases, where surgical resection poses significant risks. Other treatment modalities, including systemic chemotherapy or conventional external beam radiation therapy, were considered less effective for this isolated pelvic metastasis. Furthermore, the patient showed a significant decline in AFP levels post-treatment, highlighting the efficacy of this approach. Previous studies have demonstrated the effectiveness of radioactive seed implantation in managing metastatic HCC, achieving improved local control rates and better tolerance compared to systemic therapies.

## Patient introduction

The patient is a 68-year-old female who was found to have elevated AFP levels during a routine examination. On November 3, 2022, her AFP was 259.8 ng/mL, and abnormal prothrombin II was 22.0 mAU/mL. The ultra-sensitive hepatitis B DNA quantification was < 10 IU/mL. A CT scan revealed a significant lesion in the liver. During follow-up, AFP levels continued to rise, and a re-examination in March 2023 showed further elevation of AFP. The patient underwent transcatheter arterial chemoembolization (TACE), but her AFP levels remained persistently elevated post-procedure. On March 1, 2024, the patient underwent liver tumor resection due to the presence of a hepatic mass. Postoperative pathology indicated moderately to poorly differentiated HCC with invasion of the liver capsule. However, AFP levels continued to rise, reaching 2751 ng/mL on March 20, 2023. By May 10, 2024, AFP levels had escalated to 15930.0 ng/mL. A PET-CT scan performed on May 20, 2024, revealed the following: 1. Post-resection changes in the left hepatic lobe with no significant abnormal glucose metabolism in the surgical area; an irregular soft tissue mass with increased glucose metabolism in the recto-uterine pouch, suggestive of metastasis, though other possibilities could not be ruled out—biopsy was recommended for confirmation. Additionally, a soft tissue density nodule in the right retroperitoneal space and small nodules in both lungs were observed, with no significant abnormal glucose metabolism, warranting close follow-up with CT to exclude metastasis. 2. A mixed-density nodule in hepatic segment VII with glucose metabolism lower than that of the liver, along with punctate dense shadows within the liver parenchyma—further clinical history and other imaging studies were recommended for correlation; a liver cyst, mild dilation of the bile ducts at the hepatic hilum, and common bile duct were also noted. 3. Postoperative changes in the abdominal wall.

**Figure 2:**
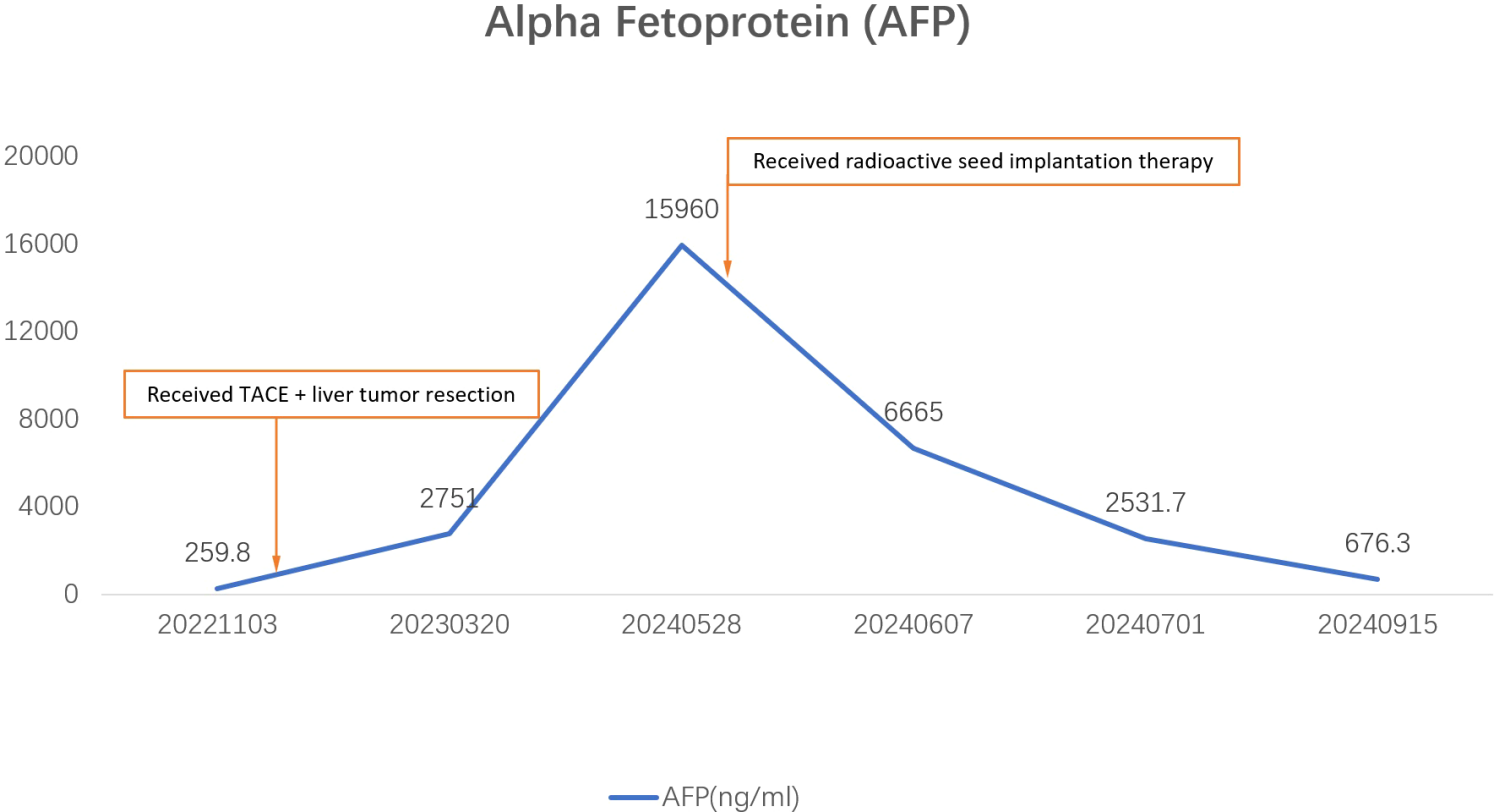
Line chart showing changes in alpha-fetoprotein (AFP) levels during the patient’s treatment. The graph indicates that AFP levels did not improve after treatment of the primary liver tumor but significantly decreased following radioactive seed implantation therapy for the pelvic metastatic lesion.

On May 15, 2024, the patient underwent a percutaneous biopsy of the irregular soft tissue in the recto-uterine pouch, which confirmed the presence of a HCC metastasis. The patient had no history of hepatitis, tuberculosis, hypertension, heart disease, trauma, or blood transfusions. On May 29, 2024, the patient underwent radioactive seed implantation for the metastatic tumor in the recto-uterine pouch. The procedure was uneventful, with no bleeding, infection, hematuria, or rectal and bladder irritation symptoms. Follow-up AFP levels were 6665.0 ng/mL at 4 weeks post-procedure and 2531.7 ng/mL at 6 weeks post-procedure, with abnormal prothrombin II remaining at 22.0 mAU/mL (**Figure 2**).

## Discussion

Metastatic lesions of HCC typically resemble the primary tumor in both structure and biological characteristics. In HCC patients, the most common site of metastasis is within the liver, though extrahepatic metastasis can also occur, albeit less frequently. Common sites of extrahepatic metastasis include the lungs, peritoneum, bones, subphrenic lymph nodes, spleen, and adrenal glands (9, 10). Aside from the lungs, these extrahepatic metastases are generally located in the upper abdomen. Bone metastases most frequently occur in the vertebrae, while peritoneal dissemination typically presents as multifocal lesions (11). However, isolated pelvic metastasis is exceedingly rare in HCC.

Studies have shown that the cumulative incidence of isolated pelvic metastasis within three years in HCC patients is only 1.4% (95% CI: 0.7, 2.2), with the incidence of incidental pelvic tumors being even lower at just 0.5% (95% CI: 0.1, 1.0) (12). Research by Szklaruk et al. (13) further explored the prevalence of pelvic lesions in HCC patients, finding that approximately 5% of patients had new lesions in the pelvic region, with a 3% incidence of isolated pelvic metastasis. Isolated pelvic metastasis in HCC patients, such as the case presented in this report, is exceedingly rare, with an estimated cumulative incidence of only 1.4% within three years. In contrast, other more common sites of HCC metastasis have been identified in large-scale epidemiological studies. According to a nationwide study, the lungs are the most frequent site of extrahepatic metastasis (31% of cases), followed by the peritoneum (3%) and bones (3%) (9). Less common sites include lymph nodes, spleen, and adrenal glands, each accounting for approximately 1% of cases. Metastases to regions such as the brain, kidneys, and ovaries are exceedingly rare, each comprising less than 0.5% of cases. The pelvic region, specifically the recto-uterine pouch, poses unique diagnostic challenges due to its deep anatomical location and less pronounced vascularization compared to other metastatic sites. These factors may explain the slower tumor growth and atypical clinical presentation observed in this case. This highlights the importance of considering rare metastatic sites in the differential diagnosis, particularly in patients with persistently elevated AFP levels but no evident metastasis in more common sites on imaging. Furthermore, the findings of this study underscore the critical role of advanced imaging modalities and pathological confirmation in diagnosing and managing these rare cases.

Patients with declining or negative AFP levels in HCC tend to have relatively better prognoses. Several reasons explain this observation: 1. Studies have shown that AFP is primarily located on the cell surface or within the cytoplasm. In the cytoplasm, AFP is internalized into cells through AFP receptors (14). This process relies on the cAMP-PKA signaling pathway and Ca2+ influx to promote tumor proliferation, leading to an increase in intracellular Ca2+ and a corresponding rise in cAMP, which activates protein kinase A (PKA). This activation enhances DNA synthesis, thus facilitating tumor cell proliferation. 2. Research has found that AFP can stimulate the expression of various oncogenes involved in cell proliferation, including c-fos, c-jun, and N-ras, and upregulate proteins involved in cell cycle progression, as well as stimulate endothelial cell proliferation induced by vascular endothelial growth factor (VEGF) (15). Moreover, AFP can interact with caspases, blocking the apoptosis of HCC cells. To further confirm the growth-promoting role of AFP, Yang et al. (16) silenced AFP expression in HCC cell lines, resulting in inhibited cell proliferation and increased apoptosis. 3. In patients with AFP-strongly positive HCC, the risks of vascular invasion and distant metastasis are significantly higher. Studies have shown that AFP-positive HCC patients have a higher proportion of poorly differentiated tumors and vascular invasion compared to AFP-negative patients, which inevitably leads to a poorer prognosis for AFP-positive patients (17). 4. Song et al. (18) found that the proportion of cirrhosis and patients in Barcelona Clinic Liver Cancer (BCLC) stages B and C was significantly higher in the AFP-positive group compared to the AFP-negative group, suggesting that AFP-positive patients have a poorer hepatic background. The degree of cirrhosis is a crucial factor affecting the prognosis of HCC, and poor liver function also contributes to greater surgical difficulty and poorer outcomes. This observation further underscores the critical role of monitoring AFP levels postoperatively. Consistent surveillance of AFP not only aids in detecting potential recurrence or metastasis but also provides valuable insights into the overall disease activity and treatment efficacy in HCC patients. In summary, AFP-negative HCC patients generally have better overall prognoses than AFP-positive patients, which includes factors such as lower risks of vascular invasion and better liver function status.

With increasing understanding of AFP, the role of AFP in evaluating the surgical prognosis of HCC has become controversial. Some researchers believe that AFP levels are negatively correlated with surgical prognosis, meaning that higher preoperative AFP levels are associated with lower overall survival rates and disease-free survival rates post-surgery, indicating poorer prognosis (19, 20). Yang et al. (16) suggest that AFP levels are closely related to long-term prognosis; higher AFP levels are associated with lower survival rates and higher recurrence rates, particularly for long-term survival. Meta-analysis results show that the disease-free survival rate in the AFP-negative group is significantly higher than that in the AFP-positive group (21). The likelihood of primary HCC metastasizing is relatively high and generally shares the same pathological characteristics as the primary lesion. While many studies have shown that elevated AFP levels are associated with poorer overall and disease-free survival, other research suggests that AFP may not be a definitive prognostic marker. For instance, Some researchers believe that AFP levels alone are insufficient to predict outcomes (22, 23), as factors such as tumor size, vascular invasion, and hepatic function play equally significant roles. Furthermore, it has been noted that some AFP-negative patients still exhibit aggressive tumor behavior, challenging the reliance on AFP as a sole prognostic indicator. This highlights the need for a more comprehensive approach, integrating AFP with other clinical and pathological parameters to improve prognostic accuracy. In this case, after resection of the intrahepatic primary lesion, AFP levels did not decrease and continued to rise. However, after treatment of the rectal uterine cul-de-sac lesion, AFP significantly decreased, suggesting that AFP was primarily caused by the metastatic lesions. The metastatic lesions were highly active, with rapid tumor cell proliferation, but were small and difficult to detect, indicating the necessity of PET-CT for examination. In addition to AFP, prothrombin II (PIVKA-II) has emerged as a valuable biomarker for HCC diagnosis and monitoring. PIVKA-II reflects tumor-induced alterations in vitamin K metabolism and is particularly useful in cases where AFP levels remain within the normal range (23). Studies have shown that combining AFP and PIVKA-II improves diagnostic sensitivity and specificity, enabling more accurate detection of HCC. In this case, elevated PIVKA-II levels (22.0 mAU/mL) further supported the diagnosis of HCC and correlated with tumor activity, underscoring its utility in clinical practice. Future studies are warranted to explore the prognostic implications of PIVKA-II in monitoring treatment response and predicting outcomes in HCC patients.

In summary, isolated pelvic metastases and incidental pelvic tumors are relatively rare in HCC patients. Future research is needed to explore optimal pelvic scanning strategies and the impact of pelvic CT coverage on the survival of HCC patients. The importance of AFP monitoring is supported by clinical guidelines, which recommend regular follow-ups to improve long-term outcomes in HCC patients (24, 25).

## Data Availability

Due to ethical concerns and to protect patient confidentiality, the data are not publicly available. Access can be granted upon reasonable request and approval by the corresponding author.
